# Item

**Published:** 1905-06

**Authors:** 


### ITEM.

### American Medical Association—Rates to Portland.

A CIRCULAR in relation to the meeting of the American Medical Association at Portland, Ore., July 11-14, 1905, has been addressed to the medical profession, concerning rates, routes and other similar points of importance. It reads as follows:

In order that the trip to the meeting of the American Medical Association at Portland, Ore., this year, may be enjoyed by the largest possible number of physicians and surgeons, and particularly by those whose professional duties make it necessary that the journey be performed in an expeditious manner, a special overland limited train has been arranged for, and a special party is being organised to leave Chicago via the Chicago, Union Pacific and Northwestern Line the evening of July 6, to make the trip to Portland surrounded by every luxury that modern travel can provide, upon schedules occupying a little less than seventy hours en route.

It is not often that so congenial a party as this is available, traveling under such highly favorable surroundings for comfort and enjoyment of the trip. A description of the train and route is included in the attached circular, and you are most cordially invited to become a member of the party.

Arrangements have been made with the railways for the movement of this special overland limited train without any extra charge for the unusual service thus secured.

The round-trip rate Chicago to Portland and return is $56.50, and proportionately low rates from other points will be in effect. Ask your ticket agent for your tickets via the Chicago & Northwestern and Union Pacific lines. Return trip may be made via Oregon Short Line, Salt Lake City, and through Colorado without extra charge, or via the Northern Pacific, Great Northern or Canadian Pacific without extra charge, or via San Francisco and Los Angeles on the payment of $11.00 additional at time of purchase. Sleeping car rate Chicago to Portland for double berth in special train, $14.00. Drawing room, $53.00. Stop-overs will be permitted at and west of Colorado common points, Cheyenne or Trinidad, inclusive, or Saint Paul and Minneapolis and a tour of the Yellowstone Park may be made either via Monida or Livingston at a rate of $49.50 additional, covering the usual tour through the park, including stage transportation and hotel accommodations.

Will you please, upon receipt, advise Mr. H. A. Gross, General Agent, Passenger Department, Chicago & Northwestern Railway, 212 Clark street, Chicago, Ill., or the nearest representative whose name and address appears on the accompanying circular, whether you desire to be included in the party, and how much sleeping car space you desire reserved?

Further information in regard to the trip will be cheerfully furnished on application.

S. C. Stanton, M.D., Room 1609, 59 State St., Chicago, Ill.
Heinrich Stern, M.D., 56 East 76th St., New York, N. Y.
Lewis S. McMurtry, M.D., 1912 Sixth St., Louisville, Ky.
Wm. Warren Potter, M.D., 284 Franklin St., Buffalo, N.Y.
Florus F. Lawrence, M.D., Columbus, O.
Simon P. Scherer, M.D., Indianapolis, Ind.
Charles A. L. Reed, M.D., The Groton, Cincinnati, O.

